# Evaluating the relationship between circulating lipoprotein lipids and apolipoproteins with risk of coronary heart disease: A multivariable Mendelian randomisation analysis

**DOI:** 10.1371/journal.pmed.1003062

**Published:** 2020-03-23

**Authors:** Tom G. Richardson, Eleanor Sanderson, Tom M. Palmer, Mika Ala-Korpela, Brian A. Ference, George Davey Smith, Michael V. Holmes

**Affiliations:** 1 Medical Research Council Integrative Epidemiology Unit, University of Bristol, Bristol, United Kingdom; 2 Population Health Sciences, Bristol Medical School, University of Bristol, Barley House, Oakfield Grove, Bristol, United Kingdom; 3 Systems Epidemiology, Baker Heart and Diabetes Institute, Melbourne, Australia; 4 Computational Medicine, Faculty of Medicine, University of Oulu and Biocenter Oulu, Oulu, Finland; 5 NMR Metabolomics Laboratory, School of Pharmacy, University of Eastern Finland, Kuopio, Finland; 6 Department of Epidemiology and Preventive Medicine, School of Public Health and Preventive Medicine, Faculty of Medicine, Nursing and Health Sciences, The Alfred Hospital, Monash University, Melbourne, Australia; 7 Centre for Naturally Randomized Trials, University of Cambridge, Cambridge, United Kingdom; 8 MRC/BHF Cardiovascular Epidemiology Unit, Department of Public Health and Primary Care, University of Cambridge, Cambridge, United Kingdom; 9 Medical Research Council Population Health Research Unit, University of Oxford, Oxford, United Kingdom; 10 Clinical Trial Service Unit & Epidemiological Studies Unit, Nuffield Department of Population Health, University of Oxford, Oxford, United Kingdom; University of Pennsylvania, UNITED STATES

## Abstract

**Background:**

Circulating lipoprotein lipids cause coronary heart disease (CHD). However, the precise way in which one or more lipoprotein lipid-related entities account for this relationship remains unclear. Using genetic instruments for lipoprotein lipid traits implemented through multivariable Mendelian randomisation (MR), we sought to compare their causal roles in the aetiology of CHD.

**Methods and findings:**

We conducted a genome-wide association study (GWAS) of circulating non-fasted lipoprotein lipid traits in the UK Biobank (UKBB) for low-density lipoprotein (LDL) cholesterol, triglycerides, and apolipoprotein B to identify lipid-associated single nucleotide polymorphisms (SNPs). Using data from CARDIoGRAMplusC4D for CHD (consisting of 60,801 cases and 123,504 controls), we performed univariable and multivariable MR analyses. Similar GWAS and MR analyses were conducted for high-density lipoprotein (HDL) cholesterol and apolipoprotein A-I. The GWAS of lipids and apolipoproteins in the UKBB included between 393,193 and 441,016 individuals in whom the mean age was 56.9 y (range 39–73 y) and of whom 54.2% were women. The mean (standard deviation) lipid concentrations were LDL cholesterol 3.57 (0.87) mmol/L and HDL cholesterol 1.45 (0.38) mmol/L, and the median triglycerides was 1.50 (IQR = 1.11) mmol/L. The mean (standard deviation) values for apolipoproteins B and A-I were 1.03 (0.24) g/L and 1.54 (0.27) g/L, respectively. The GWAS identified multiple independent SNPs associated at *P* < 5 × 10^−8^ for LDL cholesterol (220), apolipoprotein B (*n* = 255), triglycerides (440), HDL cholesterol (534), and apolipoprotein A-I (440). Between 56%–93% of SNPs identified for each lipid trait had not been previously reported in large-scale GWASs. Almost half (46%) of these SNPs were associated at *P* < 5 × 10^−8^ with more than one lipid-related trait. Assessed individually using MR, LDL cholesterol (odds ratio [OR] 1.66 per 1-standard-deviation–higher trait; 95% CI: 1.49–1.86; *P* < 0.001), triglycerides (OR 1.34; 95% CI: 1.25–1.44; *P* < 0.001) and apolipoprotein B (OR 1.73; 95% CI: 1.56–1.91; *P* < 0.001) had effect estimates consistent with a higher risk of CHD. In multivariable MR, only apolipoprotein B (OR 1.92; 95% CI: 1.31–2.81; *P* < 0.001) retained a robust effect, with the estimate for LDL cholesterol (OR 0.85; 95% CI: 0.57–1.27; *P* = 0.44) reversing and that of triglycerides (OR 1.12; 95% CI: 1.02–1.23; *P* = 0.01) becoming weaker. Individual MR analyses showed a 1-standard-deviation–higher HDL cholesterol (OR 0.80; 95% CI: 0.75–0.86; *P* < 0.001) and apolipoprotein A-I (OR 0.83; 95% CI: 0.77–0.89; *P* < 0.001) to lower the risk of CHD, but these effect estimates attenuated substantially to the null on accounting for apolipoprotein B. A limitation is that, owing to the nature of lipoprotein metabolism, measures related to the composition of lipoprotein particles are highly correlated, creating a challenge in making exclusive interpretations on causation of individual components.

**Conclusions:**

These findings suggest that apolipoprotein B is the predominant trait that accounts for the aetiological relationship of lipoprotein lipids with risk of CHD.

## Author summary

**Why was this study done?:**

- There is uncertainty regarding which lipid or apolipoprotein trait is the predominant atherogenic agent involved in the aetiology of lipids and coronary heart disease (CHD).
- The elucidation of such is important because not only does it clarify the aetiological understanding of the pathogenesis of CHD, it also hones the focus on the lipid- or apolipoprotein-related trait that should be the focus when developing lipid-modifying interventions.

**What did the researchers do and find?:**

- We used data on up to 441,016 participants from the UK Biobank to conduct genome-wide association analyses to find genetic variants reliably associated with lipoprotein lipid and apolipoprotein concentrations: this led to the identification of multiple independent genetic variants, each associated very robustly (at *P* < 5 × 10^−8^) with LDL cholesterol (220 genetic variants), apolipoprotein B (255 genetic variants), triglycerides (440 genetic variants), HDL cholesterol (534 genetic variants), and apolipoprotein A–I (440 genetic variants). Hundreds of these variants identified were novel, to our knowledge.
- When we explored the potential causal role of these lipids and apolipoproteins in isolation using Mendelian randomisation, we found evidence compatible with LDL cholesterol, triglycerides, and apolipoprotein B increasing the risk of CHD and HDL cholesterol and apolipoproteins A-I lowering CHD risk.
- When we examined these lipids and apolipoproteins together in multivariable Mendelian randomisation, we found that only apolipoprotein B retained a robust relationship with the risk of CHD—the effect estimates for all other entities were either substantially attenuated to the null or reversed in direction.

**What do these findings mean?:**

- The analytical approach in this study implemented through multivariable Mendelian randomisation simultaneously accounts for genetic associations with lipids and apolipoproteins and should therefore provide more reliable insights into what is the underlying driver of CHD.
- These findings support that, amongst the repertoire of lipoprotein lipids and apolipoproteins that we investigated, apolipoprotein B has a fundamental role in the aetiology of CHD.

## Introduction

There is incontrovertible evidence that lipids play a causal role in the aetiology of coronary heart disease (CHD) [1–3]. Multiple large-scale randomised trials of lipid-modifying therapies have conclusively shown that lowering of cholesterol in atherogenic lipoproteins leads to a reduction in risk of CHD [4,5]. These findings have been recapitulated in human genetic studies using genetic variants robustly associated with low-density lipoprotein (LDL) cholesterol [6–9].

Each circulating atherogenic lipoprotein particle includes one apolipoprotein B molecule, but the amount of cholesterol (especially in LDL particles) and the amount of triglycerides (especially in very low-density lipoprotein [VLDL] particles) can vary extensively between lipoprotein particles [10–12]. Thus, whilst the concentration of LDL cholesterol and triglycerides quantifies the concentration of these lipid substances carried in circulating lipoproteins, they do not precisely quantify the number of atherogenic lipoproteins; in contrast, the concentration of apolipoprotein B molecules is directly proportional to the number of circulating atherogenic particles in the blood [11,12]. Evidence from human genetics supports a causal role of LDL cholesterol, triglycerides, and apolipoprotein B in CHD [13–16]. Whilst it is plausible that each lipid-related entity does individually play a causal role, it is also feasible that one trait predominates and ultimately accounts for the relationship of lipoprotein particle entities. Elucidating the comparative role of lipoprotein lipids in the aetiology of CHD has important repercussions not only in terms of a clearer understanding of the underlying pathophysiology but also in terms of which biomarker(s) should be the focus of lipid-modifying therapeutics, could provide the best surrogate endpoint for honing down to the most effective agents to enter into event trials, and might have more application in the clinical setting.

Disentangling the relationships of atherogenic lipoprotein lipids and risk of CHD is nontrivial, given the correlated nature of these traits [17,18]. From the perspective of which trait has the strongest association with cardiovascular risk, meta-analyses have identified that apolipoprotein B has a comparatively higher relative risk than that derived from LDL cholesterol [19]. The inclusion of apolipoprotein-B–related measures leads to modest incremental increases in predictive performance [20]. Pooled data from over 500,000 individuals across 44 cohorts identified non-high–density lipoprotein (non-HDL) cholesterol (an approximation to the number of apolipoprotein-B–containing particles) to strongly associate with risk of incident cardiovascular disease [21]. When the concentrations of apolipoprotein B and LDL cholesterol are discordant in individuals, apolipoprotein B has a stronger association with risk of cardiovascular disease (CVD) than LDL cholesterol [22]. This is of clinical relevance given that it is estimated that more than one-quarter of the general population has meaningful discordant apolipoprotein B and LDL cholesterol values, with greater evidence of such discordance in individuals with metabolic risk factors such as obesity or type 2 diabetes [23]. Notably, the 2018 American College of Cardiology/American Heart Association (ACC/AHA) guidelines from the US [24] do not recommend routine measurement of apolipoprotein B in risk prediction, whereas the 2019 European guidelines from the European Society of Cardiology/European Atherosclerosis Society (ESC/EAS) [25] do recommend that all persons should have apolipoprotein B measured, if available.

In genetic analyses, a similar ‘discordance’ analytical approach can help to elucidate the comparative causal roles of lipid-related traits on CHD through the selection of genetic variants that associate with more than one trait. This can help identify which of the traits has a predominant influence on risk of CHD. Such studies have identified that, when genetic associations with LDL cholesterol or triglycerides are discordant with their association with apolipoprotein B, it is apolipoprotein B that retains the most robust relationship. [15,26] Another approach is to use Mendelian randomisation (MR), a genetic approach that can facilitate an assessment of causality under certain assumptions. [27] Conventionally, MR involves the analysis of individual exposure to outcome relationships. A recently developed extension to MR, so-called multivariable MR, permits the appraisal of multiple risk factors simultaneously. By including the genetic associations for multiple exposures in the same model, multivariable MR can elucidate which traits retain a causal relationship with an outcome of interest through the genetic protection against conventional confounders including reverse causation, the inherent correction for measurement error, and the avoidance of collider bias [28].

In this study, we sought to use human genetics to disentangle which one or more of the atherogenic lipid-related traits (apolipoprotein B, LDL cholesterol, and triglycerides) predominantly accounts for the causal relationship with risk of CHD. We first conducted a de novo genome-wide association study (GWAS) of lipid-related traits using the UK Biobank (UKBB) to identify variants robustly associated with each trait. We then conducted MR analyses, including multivariable MR, to elucidate which of the atherogenic lipid traits is of fundamental relevance to CHD. Finally, we investigated whether the entity underlying the causal role of atherogenic lipid-related traits in CHD also accounted for the inverse association of HDL-related phenotypes with CHD.

## Methods

### Data sources

We used data from the UK Biobank (UKBB) under application #15825 and summary estimates from CARDIoGRAMplusC4D [29]. Details on the UKBB, including geographical regions, recruitment processes, and other characteristics, have been previously described [30]. All individual participant data used in this study were obtained from the UKBB study, who have obtained ethics approval from the Research Ethics Committee (REC; approval number: 11/NW/0382) and informed consent from all participants enrolled in UKBB.

### Lipid traits and data handling

We explored the following traits measured in the UKBB: LDL cholesterol, apolipoprotein B, triglycerides, HDL cholesterol, and apolipoprotein A-I. Details on sample handling and assays used have been previously described [31,32].

Lipid-related traits in the UKBB were standardised/normalised using inverse rank-normalisation such that the mean was 0 and standard deviation was 1, allowing comparison of effect estimates between traits.

### GWAS of lipid-related traits in UKBB

GWAS analyses were conducted in UKBB participants of European descent based on K-means clustering (K = 4) after standard exclusions including withdrawn consent, mismatch between genetic and reported sex, and putative sex chromosome aneuploidy [33]. We identified single nucleotide polymorphisms (SNPs) associated with each of the lipid-related traits using the BOLT-LMM (linear mixed model) software[34]. Analyses were adjusted for age, sex, and a binary variable denoting the genotyping chip individuals were allocated to in UKBB (the UKBB Axiom array or the UK BiLEVE array). BOLT-LMM employs an LMM to evaluate the association between genetic variants and phenotypic traits whilst accounting for population stratification and cryptic relatedness [34]. This approach has been shown to provide higher statistical power when applied to the approximately 459,000 European samples in the UKBB study compared to alternative approaches [35]. Further details on genotyping quality control, phasing, imputation, and association testing have been reported previously [36,37]. We assigned a SNP as associated with a lipid-related trait of interest through use of a conventional GWAS threshold (*P* < 5 × 10^−8^), and SNPs were binned into loci based on pairwise linkage disequilibrium (LD; at between-SNP r^2^ < 0.001), with the SNP with the strongest association with the trait of interest (as defined by *P*-value) being retained in each locus. This process (conventionally referred to as ‘LD clumping’) was undertaken for each trait in turn using the software PLINK [38], based on a reference panel of 503 Europeans from phase 3 (version 5) of the 1,000 Genomes Project [39]. We defined novel SNPs as those associated with the trait of interest at *P* < 5 × 10^−8^ in our analyses in which an association had not been previously reported at *P* < 5 × 10^−8^, within 1 MB and at r^2^ < 0.001, by the Global Lipids Genetics Consortium [40] (for LDL cholesterol, triglycerides, and HDL cholesterol) or by Kettunen and colleagues [41] (for apolipoprotein B or apolipoprotein A-I).

### Synthesis and characterisation of genetic instruments

SNPs associating with lipid-related traits at conventional GWAS thresholds (*P* < 5 × 10^−8^) were taken forward to generate genetic instruments for each phenotype. A genetic instrument consists of one or more genetic variants that has characteristics that enable its use as an instrumental variable in MR [27,42,43]. We characterised the genetic instruments in 2 ways. First, to characterise the ‘specificity’ of individual SNPs included in each genetic instrument, we elucidated how many SNPs associated with lipid or apolipoprotein traits other than the primary lipid trait of interest at conventional GWAS thresholds of significance (*P* < 5 × 10^−8^) and used this information to generate a Venn diagram. Further LD clumping was undertaken to define the unique number of overlapping genetic loci between all 5 traits to be depicted in the Venn diagram. Second, we characterise instrument ‘specificity’ (i.e., the degree to which genetic instruments for one lipid or apolipoprotein trait also associate with other traits) by taking per-allele SNP estimates from our GWAS for each lipid trait and conducting inverse variance weighted regressions on these summary estimates to elucidate the association of genetic instruments across the various lipid-related traits—these estimates are presented as standardised differences per 1-standard-deviation–higher genetically predicted trait. Whilst we recognise that this approach may be prone to inflation (leading to potentially biased estimates owing to the derivation of genetic instruments for lipids and apolipoprotein traits occurring in the same data set as that in which their associations are subsequently estimated with other traits), the primary motivation is to characterise the associations of lipid instruments with the lipid-related traits: we do not interpret these as formal instrumental variable estimates. We also estimated the correlations between the beta coefficients for the genetic instruments used in the multivariable analyses.

### Outcome definition in CARDIoGRAMplusC4D

Cases in CARDIoGRAMplusC4D were defined as myocardial infarction, acute coronary syndrome, chronic stable angina, or coronary stenosis >50% [29], which we collectively describe in this study as CHD.

### Genetic analyses to elucidate potential causality

We first conducted univariable MR analyses for each lipid-related trait. For this, we harmonised SNPs identified from our GWASs of lipoprotein lipid traits in the UKBB to those SNPs available in CARDIoGRAMplusC4D by either matching the SNP directly or by selecting proxy SNPs in high LD (r^2^ > 0.8). This led to a small drop in the number of SNPs being available for MR, with a median of 93% SNPs identified in GWASs available for MR (the numbers available for each trait are provided in Table 1). We used the inverse variance weighted approach, which, in brief, takes the form of a linear regression of the SNP–outcome association regressed on the SNP–exposure association weighted by the inverse of the square of the standard error of the SNP–outcome association, with the intercept constrained at the origin.

**Table 1:** Genetic variants identified for each trait in the UKBB.

Trait	Number with Trait Measured in the UKBB with GWAS Genotyping	Mean Value (Standard Deviation)	Number of SNPs Identified in GWAS (*P* < 5 × 10^−8^)	Number (%) of Novel* SNPs	Number (%) of SNPs Aligned to CARDIoGRAM-plusC4D	F-statistic (Overall, Conditional)
**LDL cholesterol (mmol/L)**	440,546	3.57 (0.87)	220	123 (56%)	209 (95%)	164, 34
**Triglycerides (mmol/L)**	441,016	1.50 (1.11)^§^	440	339 (77%)	409 (93%)	116, 78
**Apolipoprotein B (g/L)**	439,214	1.03 (0.24)	255	203 (80%)	234 (92%)	153, 36^^^
**HDL cholesterol (mmol/L)**	403,943	1.45 (0.38)	534	383 (72%)	490 (92%)	124, 67
**Apolipoprotein A-I (g/L)**	393,193	1.54 (0.27)	440	407 (93%)	407 (93%)	120, 62

*We defined novel SNPs as those associated with the trait of interest at *P* < 5 × 10^−8^ in which an association had not been previously reported at *P* < 5 × 10^−8^, within 1 MB and at r^2^ < 0.001 by the Global Lipids Genetics Consortium [40] (for LDL cholesterol, triglycerides and HDL cholesterol) or by Kettunen and colleagues [41] (for apolipoprotein B or apolipoprotein A-I).

^§^Median (IQR) presented for triglycerides, owing to the non-Gaussian distribution.

^^^The conditional F-statistic for apolipoprotein B when included in the multivariable MR model with LDL cholesterol and triglycerides was 36, and in the multivariable MR model that included HDL cholesterol and apolipoprotein A-I, it was 66.

**Abbreviations:** GWAS, genome-wide association study; HDL, high-density lipoprotein; IQR, interquartile range; LDL, low-density lipoprotein; MR, Mendelian randomisation; SNP, single nucleotide polymorphism; UKBB, UK Biobank.

We next conducted multivariable MR, which is a statistical approach that allows for the association of SNPs with multiple phenotypes to be incorporated into the analysis, permitting an estimation of the direct effect of each phenotype on the outcome (i.e., an effect that is not mediated by any other factor in the model [28]); see S1 Fig for further details. In this manuscript, we use the term ‘adjusted’ in the context of multivariable MR to mean ‘direct’ effects, i.e., the effect of a lipid trait on CHD whilst accounting for either mediation or confounding by another trait included in the model. For the multivariable MR analyses, we fitted a model with apolipoprotein B, LDL cholesterol, and triglycerides to identify which one or more traits appeared to be responsible for the effect of ‘atherogenic’ lipid-related traits on risk of CHD. We then took the atherogenic trait(s) that retained an effect on CHD in the multivariable MR model forward and further adjusted for apolipoprotein A-I and HDL cholesterol to assess the potential causal roles of HDL-related phenotypes in the development of CHD. In the setting of multivariable MR, we included all GWAS-associated SNPs for all traits in the model. This meant that there were differing numbers of SNPs in the 2 multivariable models tested.

We characterised instrument strengths in both the univariable and multivariable MR settings as follows: for the univariable estimates, we generated the mean F-statistic, using the approximation described by Bowden and colleagues [44]. For the multivariable estimate, we generated the conditional F-statistic [28,45]. Further details are provided in S1 Text.

### Software

The BOLT-LMM software was used to undertake GWAS [34]. This does not require the use of principal components in GWAS to account for population structure because this is taken into account using an LMM as performed by the software [35]. MR analyses were conducted using the ‘TwoSampleMR’ R package [46]. Manhattan and forest plots were generated using the ‘ggplot2’ and ‘metafor’ packages, respectively [47,48].The Venn diagram was generated using the online tool available at http://bioinformatics.psb.ugent.be/webtools/Venn (accessed 13th August 2019).

### Interpretation of findings

Whilst we desisted from dichotomising results of analyses purely on the basis of a *P*-value into being ‘significant’ or not [49,50], as a means of grading the strength of evidence against the null hypothesis, in both the univariable and multivariable MR analyses, we used a two-sided alpha of 0.01 on the basis of testing 5 lipid-related traits. Such a Bonferroni adjustment to account for multiple testing can be considered overly conservative, given the high correlation between the lipid-related traits.

### Sensitivity analyses

In sensitivity analyses, we conducted univariable MR analyses robust to some forms of potential unbalanced horizontal pleiotropy [51] (horizontal pleiotropy being the process by which genetic variants used to instrument an exposure also associate with other traits that influence the outcome, a form of violation of the exclusion restriction assumption of instrumental variable analyses [52]) using weighted median [53], weighted mode [54], and MR-Egger [55] approaches. To further account for potential unbalanced horizontal pleiotropy in the multivariable MR analysis, we also conducted multivariable MR-Egger analyses [56,57]. To identify whether multivariable MR estimates derived from our de novo GWAS are comparable to those previously published [13] using prior GWAS consortia data for lipoprotein lipids [40], we repeated the analyses excluding apolipoprotein B from the models. Finally, we adjusted for fasting time in the derivation of the genetic instruments (by including fasting hours as a covariate in the GWAS). This is because fasting status might impact the lipid or lipoprotein concentrations [58], with a potential effect on the SNP–lipid trait associations, thus potentially influencing the MR estimates.

### Protocol

Our study did not have a prospective protocol or analysis plan. Analyses were discussed in June 2019 as described above (i.e., to conduct a GWAS of lipids and apolipoproteins and then to test their causal relevance in multivariable MR). There were no data-driven changes to analyses. Following peer review, we (i) repeated the GWAS of lipids and apolipoproteins with adjustment for fasting status and repeated MR analyses using genetic instruments from such GWAS; (ii) repeated multivariable MR including LDL cholesterol or triglycerides (rather than apolipoprotein B) to demonstrate a residual effect of HDL cholesterol, in keeping with published literature; (iii) added multivariable MR-Egger as a sensitivity analysis [56]; and (iv) added phenotypic and genetic correlations between the lipid and apolipoprotein traits. We completed the STROBE checklist (S1 STROBE Checklist).

## Results

### UKBB data set

The lipid and apolipoprotein traits were measured in 393,193 to 441,016 individuals in the UKBB that have GWAS genotyping (Table 1). The mean age of study participants was 56.9 y (range 39–73 y), and 54.2% were women. The mean (standard deviation) lipid concentrations were LDL cholesterol 3.57 (0.87) mmol/L and HDL cholesterol 1.45 (0.38) mmol/L, and the median triglycerides was 1.50 (IQR = 1.11) mmol/L. The values for apolipoproteins B and A-I were 1.03 (0.24) g/L and 1.54 (0.27) g/L, respectively. Phenotypic correlations between the traits varied from Pearson’s R −0.49 (between HDL cholesterol and triglycerides) and 0.96 (between LDL cholesterol and apolipoprotein B) (S1 Table).

### GWAS of lipoprotein lipid and apolipoprotein traits

**Fig 1:**
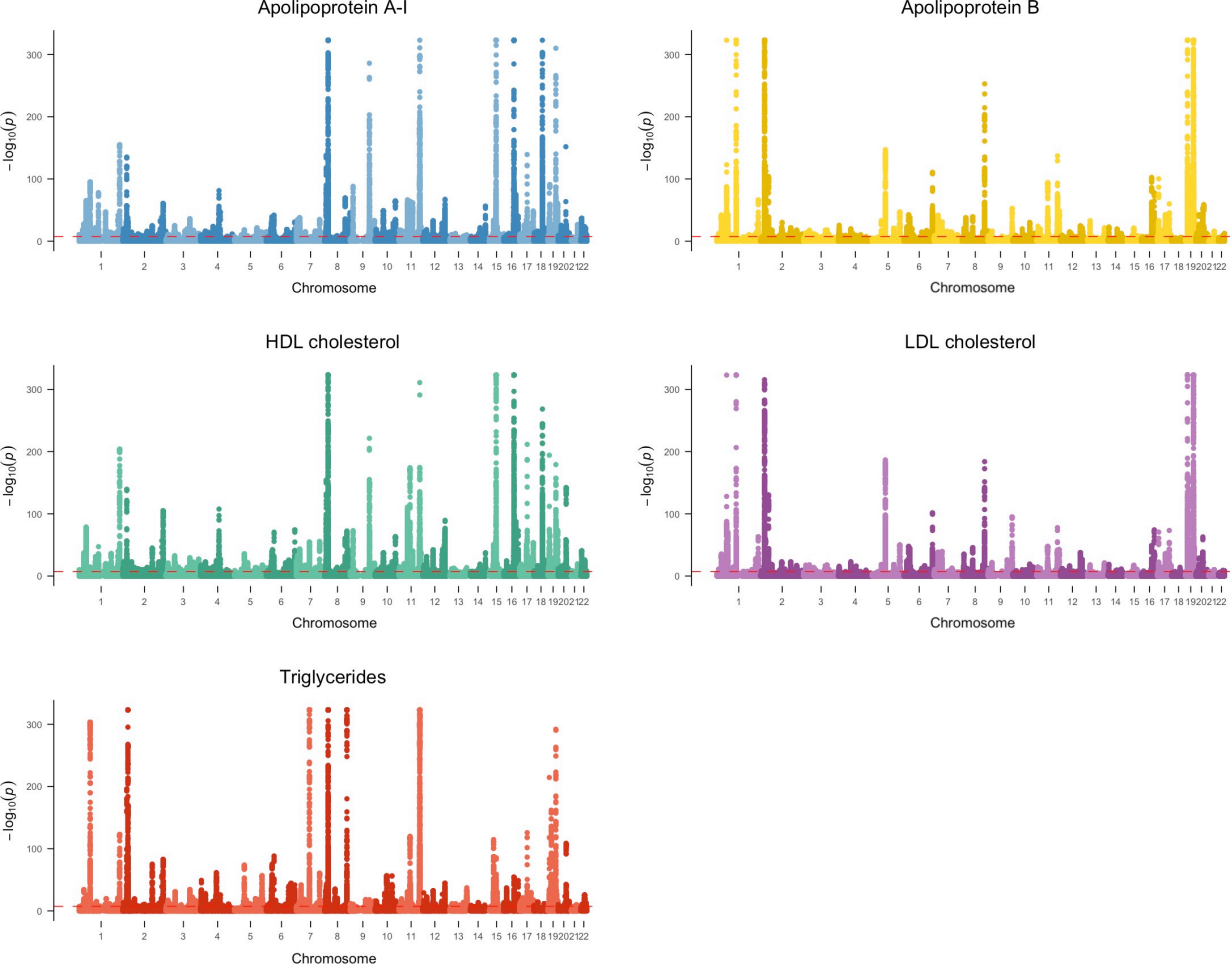
Manhattan plots showing findings from the GWAS of lipoprotein lipid-related traits in the UKBB. Horizontal dotted line illustrates the Y-axis value conventionally used to denote a SNP that reaches statistical significance in the GWAS, i.e., at *P* < 5 × 10^−8^. GWAS, genome-wide association study; HDL, high-density lipoprotein; LDL, low-density lipoprotein; SNP, single nucleotide polymorphism; UKBB, UK Biobank.

In the GWAS, we identified a large number of independent SNPs associated at *P* < 5 × 10^−8^ with each lipid-related trait: 220 SNPs (of which 56% had not been previously reported) associated with LDL cholesterol, 440 (77% novel) for triglycerides, 255 (80% novel) for apolipoprotein B, 534 (72% novel) for HDL cholesterol, and 440 (93% novel) for apolipoprotein A-I (Fig 1 and Table 1). Full details of the SNPs associated with the lipid-related traits are provided in S2–S6 Tables.

**Fig 2:**
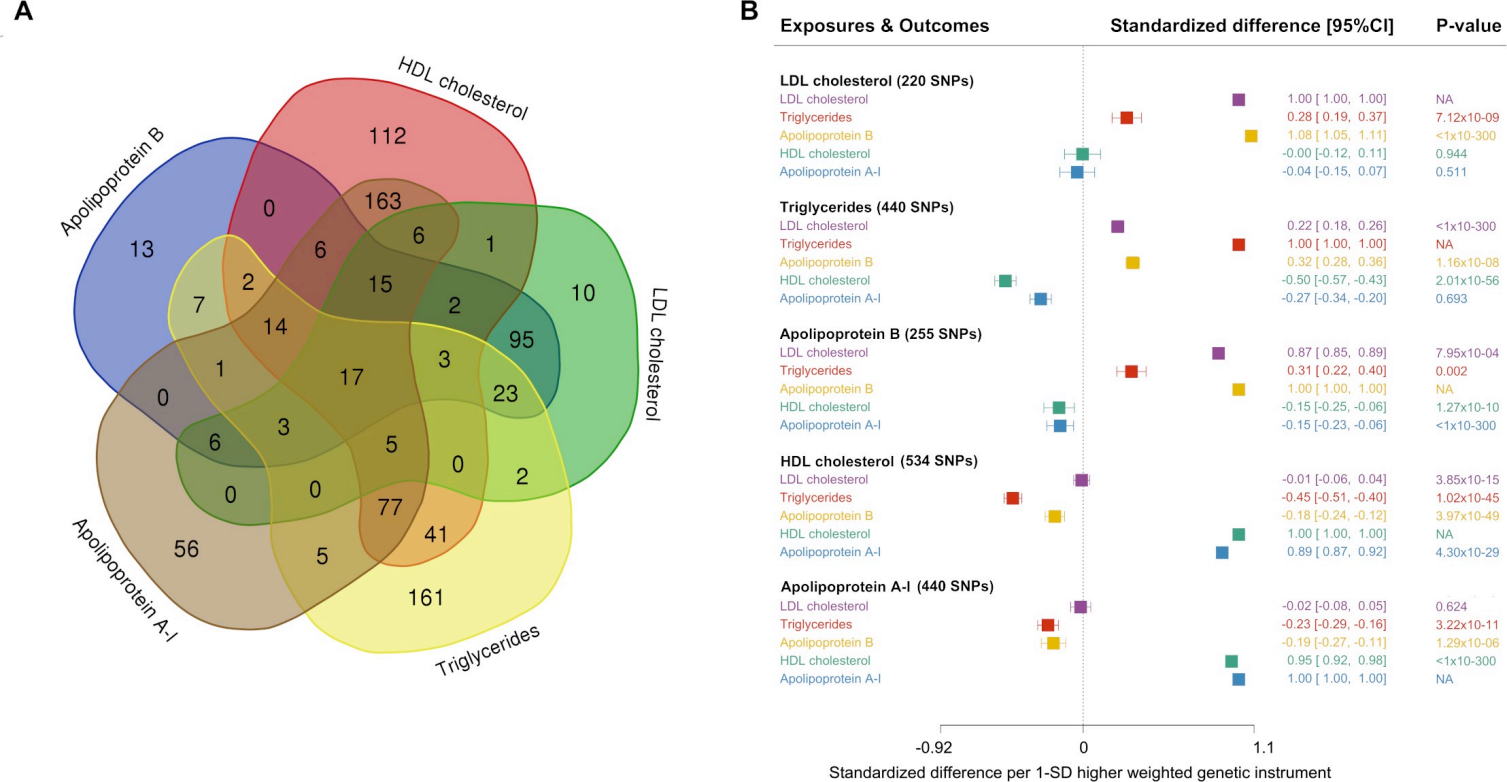
Characteristics of genetic instruments developed for lipoprotein lipid-related traits. (A) Overlap of SNPs and (B) associations with lipids and apolipoproteins. In Panel A, SNPs are grouped according to whether they associate with only the primary lipid-related trait of interest or whether they associate with other traits, based on *P* < 5 × 10^−8^. Panel B displays the associations of genetic instruments with lipid-related traits using the inverse variance weighting approach. Whilst we note the potential for overfitting of estimates displayed in Panel B, we present these data for illustrative purposes; the MR estimates presented in Fig 3 use a two-sample approach (with no overlapping data). CI, confidence interval; HDL, high-density lipoprotein; LDL, low-density lipoprotein; MR, Mendelian randomisation; NA, not applicable; SD, standard deviation; SNP, single nucleotide polymorphism.

A considerable number (352 out of a total 846 clumped SNPs, i.e., 41.6%) of SNPs used in each of the lipid-related genetic instruments showed associations at conventional GWAS significance (*P* < 5 × 10^−8^) with other lipid traits (Fig 2A). On exploring the relationships of the genetic instruments with each lipid-related trait, we identified widespread associations (Fig 2B). For example, in addition to its association with apolipoprotein B, the genetic instrument for apolipoprotein B showed strong positive associations with LDL cholesterol and triglycerides and inverse associations with HDL cholesterol and apolipoprotein A-I. Correlations between all genetic variants used as instruments ranged between Pearson’s R of −0.57 (between HDL cholesterol and triglycerides) and 0.97 (between LDL cholesterol and apolipoprotein B) (S7 Table).

**Fig 3:**
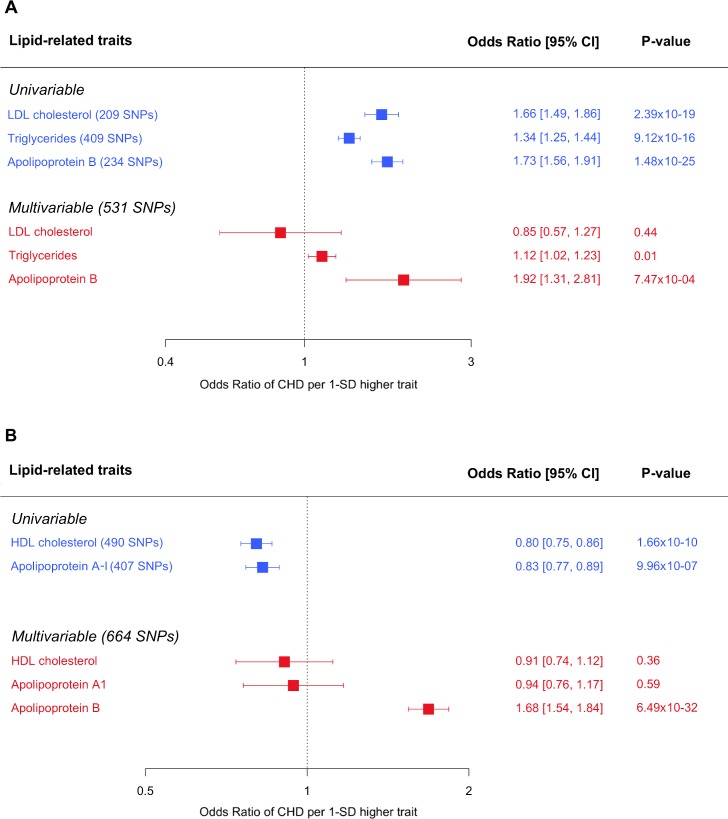
**Univariable and multivariable MR of so-called (A) atherogenic and (B) protective lipoprotein lipids and apolipoproteins and risk of CHD.** In both (A) and (B), univariable MR estimates were derived using the inverse variance weighted approach. For a more comprehensive repertoire of estimates derived from univariable MR approaches, please see S2 Fig. CHD, coronary heart disease; CI, confidence interval; HDL, high-density lipoprotein; LDL, low-density lipoprotein; MR, Mendelian randomisation; SD, standard deviation; SNP, single nucleotide polymorphism.

### Appraisal of LDL cholesterol, triglycerides, and apolipoprotein B

On individual assessment through conventional MR, we identified LDL cholesterol, triglycerides, and apolipoprotein B to have effect estimates consistent with a higher risk of CHD, using data from CARDIoGRAMplusC4D (with up to 60,801 cases) (Fig 3A). A 1-standard-deviation–higher LDL cholesterol had an odds ratio (OR) of 1.66 (95% CI: 1.49–1.86; *P* < 0.001) for CHD, with the corresponding value for triglycerides being OR 1.34, 95% CI: 1.25–1.44, and *P* < 0.001 and those for apolipoprotein B being OR 1.73, 95%CI: 1.56–1.91, and *P* < 0.001. Sensitivity analyses using methodological approaches that take into account potential genetic pleiotropy led to no substantive change in these estimates (S2 Fig).

When LDL cholesterol, triglycerides, and apolipoprotein B were assessed together in multivariable MR, only apolipoprotein B (OR 1.92; 95% CI: 1.31–2.81; *P* < 0.001) retained a robust, potentially causal relationship with CHD (Fig 3A). The point estimate for LDL cholesterol reversed on mutual adjustment to yield an adjusted OR of 0.85 (95% CI: 0.57–1.27; *P* = 0.44). The estimate for triglycerides was weakened substantially (adjusted OR 1.12; 95% CI: 1.02–1.23; *P* = 0.01).

### Appraisal of HDL cholesterol and apolipoprotein A-I

Individual analysis using conventional MR showed both HDL cholesterol and apolipoprotein A-I to have effect estimates consistent with a lower risk of CHD (Fig 3B). The estimate for a 1-standard-deviation–higher HDL cholesterol was OR 0.80 (95% CI: 0.75,–0.86; *P* < 0.001), and for apolipoprotein A-I, it was OR 0.83 (95% CI: 0.77–0.89; *P* < 0.001). The effect estimates for both HDL cholesterol and apolipoprotein A-I were diminished when using methodologies that are more robust to potential pleiotropy of the genetic variants used in the analysis (S2 Fig).

On appraisal in a multivariable MR analysis that included apolipoprotein B (which retained evidence of a potentially causal relationship with CHD on mutual genetic adjustment for LDL cholesterol and triglycerides), the effect estimates of both HDL cholesterol and apolipoprotein A-I diminished and were not distinguishable from the null (Fig 3B). The adjusted estimate for HDL cholesterol was OR 0.91 (95% CI: 0.74–1.12; *P* = 0.36), and for apolipoprotein A-I, it was OR 0.94 (95% CI: 0.76–1.17; *P* = 0.59). When adjusted for HDL cholesterol and apolipoprotein A-I, there was robust evidence for a potential causal role of apolipoprotein B in the development of CHD (adjusted OR 1.68; 95% CI: 1.54–1.84; *P* < 0.001).

The F-statistics for all lipid-related genetic instruments in both the univariable and multivariable MR settings were consistent with an absence of weak instrument bias (Table 1).

### Sensitivity analyses

Adjustment for fasting time in the GWAS of lipids and apolipoproteins led to negligible alterations in the effect estimates in both univariable and multivariable MR (S8 and S9 Tables). Use of multivariable MR-Egger led to very similar effect estimates as those derived from multivariable IVW MR (S10 and S11 Tables). Repeating the same multivariable MR models excluding apolipoprotein B led to findings similar to those identified in prior studies [13], i.e., that in multivariable MR analyses including LDL cholesterol and/or triglycerides, a weak residual inverse relationship of HDL cholesterol with CHD was seen (S12 and S13 Tables). Notably, such HDL cholesterol associations diminished to include the null on univariable MR approaches that are more robust to unbalanced horizontal pleiotropy (S2 Fig) and on multivariable MR that includes apolipoprotein B and multivariable MR-Egger (Fig 3, S10 and S11 Tables).

## Discussion

In this study, we explored the causal relationships of lipids and apolipoproteins with risk of CHD by conducting a GWAS of lipoprotein lipid-related traits in approximately 440,000 UKBB participants and taking forward genetic instruments into multivariable MR using data from the CARDIoGRAMplusC4D consortium, including over 60,000 cases of CHD. Our principal findings are that after taking into account the effects of apolipoprotein B, the relationships of other lipid-related entities with risk of CHD attenuated markedly to the null. In contrast, the relationship of apolipoprotein B with risk of CHD was robust to adjustment for other traits. Our study therefore provides empirical evidence from human genetics that apolipoprotein B is the necessary element in order for lipoprotein lipids to exert their causal effect on risk of CHD—in other words, apolipoprotein B is a critical entity that underlies the relationship of lipid traits and risk of CHD. This adds further evidence to support the hypothesis that it is the number of atherogenic lipoprotein particles indexed by apolipoprotein B rather than the amount of circulating cholesterol or triglycerides per se that is the important driver of CHD [26]. In other words, changes in cholesterol or triglycerides that are not accompanied by commensurate changes in apolipoprotein B may not lead to altered risks of CHD.

Our GWAS identified many hundreds of variants associated with the major lipid-related traits, with most SNPs identified being novel, to our knowledge. This is likely because our GWAS was not only larger in sample size than prior GWAS of lipoprotein lipid traits [40] but also used a single study. Previous GWASs [40] of lipids were conducted in multiple individual studies that were then meta-analysed, thus introducing a loss of power due to between-study heterogeneity. Many SNPs identified for one lipid-related trait also showed associations with other lipid traits, highlighting their pleiotropic nature and/or the high degree of inter-relatedness amongst plasma lipoproteins and their lipid constituents. Individual appraisal using univariable MR showed widespread effects of all lipid-related traits, with LDL cholesterol, triglycerides, and apolipoprotein B each having effect estimates consistent with a higher risk of CHD. These findings recapitulate those reported in previous studies [8,9,13,14], leading to the contemporary view that each atherogenic lipid trait might play a causal role in vascular disease. When we estimated the direct (i.e., adjusted) effect of these traits using multivariable MR (see S1 Fig for further details), only apolipoprotein B retained a robust effect with CHD, with the effect of LDL cholesterol being reversed and that for triglycerides being largely diminished, leaving only a weak residual effect. The apparent protective associations of HDL cholesterol and apolipoprotein A-1, present on univariable MR analyses, were also markedly attenuated when direct effects conditional on apolipoprotein B were estimated. Taken together, these findings indicate that amongst the lipid-related traits we investigated, it is apolipoprotein B, and thus the number of atherogenic lipoprotein particles, that predominates as the underlying cause of CHD.

How do these findings enhance the evidence base relating to lipid traits and vascular disease? Large-scale observational [59], interventional [4,5], and genetic [6–9] studies support LDL cholesterol as being causal in the aetiology of CHD. In recent years, genetic studies have provided evidence in support of triglycerides [13,14] also playing a causal role. Both LDL cholesterol and triglycerides are carried in atherogenic lipoproteins, each containing an apolipoprotein B molecule. Recent reviews [11,12,60,61] point to apolipoprotein B potentially being the necessary entity for atherosclerosis to occur, for example, through the ‘response to retention’ hypothesis, in which apolipoprotein-B–containing particles become trapped in the tunica intima of the arterial wall [62]. Our study builds on recent findings [26] to provide further empirical evidence that supports this hypothesis, but our findings do not discredit the causal roles that LDL cholesterol or triglycerides play in vascular disease. This is because apolipoprotein B does not occur in physiological isolation [61], but rather is always accompanied by cholesterol and triglycerides. In light of this, our findings pinpoint that it is apolipoprotein B that is necessary for lipid-mediated atherogenesis to occur. Indeed, our findings from multivariable MR are consistent with apolipoprotein B being an essential element allowing the atherogenic effects of LDL cholesterol and triglyceride to be expressed.

How do these findings aid us in the context of developing drugs that modify lipoprotein lipid concentrations and predicting their effects on risk of CHD? Drug-target MR studies show that, for example, modifying triglycerides through therapies such as angiopoietin-like proteins 3 and 4 (ANGPLT3/4) inhibition may represent an emerging approach to lowering the risk of CHD [63–65]. Our findings do not run contrary to these conclusions; rather, they shed light on whether the concentrations of cholesterol and/or triglycerides that are carried by apolipoprotein-B–containing lipoproteins contribute to risk of CHD beyond that encoded by apolipoprotein B. Based on these and recent data [15,26], the primary focus of lipid-modifying therapies ought to be the reduction in number of atherogenic lipoproteins (as measured by apolipoprotein B) rather than the reduction in cholesterol or triglycerides. This is especially the case when drugs have discrepant effects across these lipid traits [10,15,66]. Thus, in predicting the cardiovascular efficacy of a lipid-modifying therapeutic, apolipoprotein B can, all things being equal, be used as a reliable surrogate marker for the expected relative risk reduction in CHD—assuming, of course, that the drug under investigation does not display adverse events that arise either from target-mediated mechanisms or from off-target effects (notably, both can be investigated in human genetics studies [67]). In other words, our findings bolster the potential use of total circulating concentrations of apolipoprotein B to quantify the risk of CHD across a broad range of apolipoprotein-B–containing lipoprotein particles [25].

We note that this interpretation is in keeping with 2 important prior investigations that examined the concordance of CHD associations between SNPs associated with apolipoprotein B, LDL cholesterol [15], and triglycerides [26]. Indeed, one of these prior investigations conducted a form of multivariable MR analysis and obtained similar findings to those we report in the present study [26]. Importantly, the analysis that we conducted and report herein builds on these prior investigations by conducting a de novo GWAS of lipoprotein lipids and apolipoproteins in a large number of individuals and thus expanding on the numbers of SNPs used as instruments for each lipid-related trait but also including the full repertoire of GWAS-associated SNPs for apolipoprotein B. The derivation of genetic instruments from the same data set in similar numbers of individuals should facilitate a fairer representation of the genetic architecture of each of the traits included in the multivariable analyses. In addition to these methodological enhancements and replication of previous studies, our findings also demonstrate that therapies that seek to modify HDL or apolipoprotein A-I [68] will only have beneficial effects if they also lower apolipoprotein B. Their influence on apolipoprotein B is likely to account for any effects of therapies aimed at increasing HDL cholesterol or apolipoprotein A-I on the risk of CHD [66].

The findings that we make have been made available by 2 recent advances: first, the availability of large-scale lipoprotein lipid phenotyping and GWAS genotyping in the UKBB, providing sufficiently large numbers to permit identification of robust genetic variants (and therefore suitable genetic instruments) in order to conduct MR of each of the lipid-related traits. Use of a single study with similar numbers of individuals with measures available for each lipid-related trait enabled GWAS and the downstream synthesis of genetic instruments for each trait in which the genetic architecture of each phenotype ought to be similarly represented, allowing for a more rigorous comparative assessment of the traits in both the univariable and multivariable MR setting. Second, methodological developments in MR to include more than one trait (so-called multivariable MR) allows for direct effects (i.e., the effects of an exposure on disease, taking into account potential confounding and mediation by other traits) of multiple exposures to be assessed simultaneously and without the risk that this introduces forms of bias such as collider bias, in which, for example, conditioning on a potential mediator or a shared outcome can induce bias [28]. It is this methodological approach that allows the deduction that we make: that apolipoprotein B plays a critical role in the causal effects of lipid-related traits with risk of CHD. We note here an important theme that emerges: the discrepancy between our findings and those derived from other MR approaches that hitherto have been used in contemporary MR studies (reflected by the univariable MR estimates we present in S2 Fig). Approaches such as MR-Egger and weighted median MR can provide reliable evidence regarding causation even in the presence of confounding through unbalanced horizontal pleiotropy [69]. This is evidenced by the diminution of the HDL cholesterol association with risk of CHD from inverse variance weighted approaches to MR-Egger (S2 Fig). Importantly, such univariable MR approaches that are more robust to horizontal pleiotropy notably do not, with a few exceptions [56,70], allow simultaneous statistical adjustment for multiple traits. The repertoire of robust univariable MR approaches [51] that seek to act as sensitivity analyses for potential unbalanced horizontal pleiotropy each make a different series of assumptions [53]. In the situation when horizontal pleiotropy is present in a dose–response manner (i.e., on average, SNPs that associate with higher levels of the exposure of interest also associate with higher/lower degrees of horizontal pleiotropy), this violates the ‘InSIDE’ assumption [55], and the MR analyses yield biased estimates. This may be why the MR estimates for LDL cholesterol and triglycerides remain seemingly robust to MR-Egger and weighted median MR approaches. In this context, multivariable MR analysis can help when the traits included in the analysis account fully for the unbalanced, dose-related, horizontal pleiotropy. In the scenario that we investigate, apolipoprotein B may serve this role, and the multivariable MR results accounting for this support the conclusion that apolipoprotein B has a fundamental role in the atherogenic component of CHD risk.

Our study has interpretational challenges that may be conceived of as limitations. For example, a naïve interpretation of our findings would be that apolipoprotein B confounds the relationship of LDL cholesterol and triglycerides with risk of CHD, but this would be to neglect evidence gleaned over a century of scientific investigations into atherosclerosis [71]. In our interpretation, these findings from the multivariable MR, together with established physiological principles of lipoprotein structure and composition, are consistent with apolipoprotein B being a critical component in the entrapment of atherogenic lipoprotein particles in the tunica intima in order to initiate and maintain lipid accumulation in the development of atherosclerosis [11,12,60]. Thus, our findings are in keeping with established understanding of the development of atherosclerosis [72] but pinpoint the crucial role of apolipoprotein B in the pathogenesis as a key molecule facilitating the entrapment of atherogenic lipoprotein particles in the intima. In other words, our multivariable MR does not suggest that apolipoprotein B confounds the LDL cholesterol or triglycerides relationship, but rather that apolipoprotein B is critical for the atherogenic effects of lipoprotein lipids. An additional limitation is that In relation to studies of lipoprotein metabolism, a fundamental challenge is that lipoprotein metabolism is a continuum in the circulation, particularly in relation to apolipoprotein-B–containing particles that are downstream metabolic constructs of VLDL particles secreted from the liver [73]. Thus, all measures related to the composition of these particles are highly correlated [74]. This creates an inherent difficulty in making exclusive interpretations on individual structural components. Finally, the Venn diagram in Fig 2A gives the impression that SNPs appear to cluster into discrete silos based on associations with lipids and apolipoproteins. However, this pictorial illustration is based on arbitrary thresholds of association, and it would be unwise to interpret SNPs in each silo of the Venn diagram to mean exclusivity of association. To illustrate this empirically, there are 13 SNPs that appear to solely associate with apolipoprotein B in Fig 2. As illustrated in S14 Table, these 13 SNPs also associate with LDL cholesterol (with the weakest LDL-cholesterol–associating SNP doing so at *P* < 0.001), all in a directionally consistent way to their association with apolipoprotein B. Thus, whilst it may be tempting to generate instruments based on SNPs that fall into these visually discrete silos (in so-called ‘discordance analyses’), they are likely to be directionally pleiotropic. Indeed, given the physiological relationships of lipids and apolipoproteins, it could be argued that for a SNP to appear in the extremities of the Venn diagram (i.e., to be extremes in terms of their differential associate with one lipid-related trait as compared to another), such a SNP would likely possess properties that lead to this situation through pleiotropy, rendering MR estimates derived from such a SNP more vulnerable to producing misleading findings. For these reasons, we consider the findings from our multivariable MR analyses to be more trustworthy than MR analyses based on subsetting SNPs on the basis of associations with traits at arbitrarily defined *P*-value thresholds.

In conclusion, our findings demonstrate that apolipoprotein B is of critical importance in facilitating the causal effects of lipoprotein lipid traits and risk of CHD.

## Supporting information

S1 STROBE Checklist (PDF)

S1 Fig **Multivariable MR: (A) conventional MR, (B) conventional MR confounded by pleiotropy, (C) multivariable MR.** (A) In conventional MR, in the absence of confounding of the genetic instrument and with exclusion restriction, a suitably powered analysis can lead to causal deductions. (B) In this example, SNPs used in the LDL cholesterol instrument also associate with apolipoprotein B (apoB), leading to potential confounding of the estimate of LDL cholesterol and risk of CHD. (C) In multivariable MR, genetic instruments for each of the traits under investigation are used together with the corresponding associations of SNPs with each trait in the analysis. The analysis yields a causal estimate for each trait taking into account the relationship of that trait and the genetic variants with the other traits in the analysis. These figures are schematic representations and should not be interpreted as formal directed acyclic graphs. apoB, apolipoprotein B; CHD, coronary heart disease; CI, confidence interval; HDL, high-density lipoprotein; LDL, low-density lipoprotein; MR, Mendelian randomisation; SNP, single nucleotide polymorphism.
(PDF)

S2 Fig Univariable MR estimates for individual lipid and apolipoprotein traits. Effect estimates are ORs of CHD per 1-standard-deviation–higher genetically instrumented trait, using a range of univariable MR approaches (see Methods for further details). Plot generated using Stata SE 13.1 (StataCorp). CHD, coronary heart disease; CI, confidence interval; HDL, high-density lipoprotein; LDL, low-density lipoprotein; MR, Mendelian randomisation; nSNPs, number of single nucleotide polymorphisms; OR, odds ratio.
(PDF)

S1 Table Phenotypic correlations between lipid-related traits in UKBB. HDL, high-density lipoprotein; LDL, low-density lipoprotein; UKBB, UK Biobank.
(XLSX)

S2 Table GWAS results for LDL cholesterol. Beta, regression coefficient; BP, base position; CHR, chromosome; EAF, effect allele frequency; Gene, nearest gene; GWAS, genome-wide association study; INFO, imputation score; LDL, low-density lipoprotein; Novel signal?, association with *P* < 5 × 10^−8^ not previously detected by the Global Lipids Genetics Consortium [40] based on <1 mb and r^2^ < 0.001; P, *P*-value; SE, standard error of regression coefficient; SNP, single nucleotide polymorphism.
(XLSX)

S3 Table GWAS results for triglycerides. Beta, regression coefficient; BP, base position; CHR, chromosome; EAF, effect allele frequency; Gene, nearest gene; GWAS, genome-wide association study; INFO, imputation score; Novel signal?, association with *P* < 5 × 10^−8^ not previously detected by the Global Lipids Genetics Consortium [40] based on <1 mb and r^2^ < 0.001; P, *P*-value; SE, standard error of regression coefficient; SNP, single nucleotide polymorphism.
(XLSX)

S4 Table GWAS results for apolipoprotein B. Beta, regression coefficient; BP, base position; CHR, chromosome; EAF, effect allele frequency; Gene, nearest gene; GWAS, genome-wide association study; INFO, imputation score; Novel signal?, association with *P* < 5 × 10^−8^ not previously detected by the Global Lipids Genetics Consortium [40] based on <1 mb and r^2^ < 0.001; P, *P*-value; SE, standard error of regression coefficient; SNP, single nucleotide polymorphism.
(XLSX)

S5 Table GWAS results for HDL cholesterol. Beta, regression coefficient; BP, base position; CHR, chromosome; EAF, effect allele frequency; Gene, nearest gene; GWAS, genome-wide association study; HDL, high-density lipoprotein INFO, imputation score; Novel signal?, association with *P* < 5 × 10^−8^ not previously detected by the Global Lipids Genetics Consortium [40] based on <1 mb and r^2^ < 0.001; P, *P*-value; SE, standard error of regression coefficient; SNP, single nucleotide polymorphism.
(XLSX)

S6 Table GWAS results for apolipoprotein A-I. Beta, regression coefficient; BP, base position; CHR, chromosome; EAF, effect allele frequency; Gene, nearest gene; GWAS, genome-wide association study; INFO, imputation score; LDL, low-density lipoprotein; Novel signal?, association with *P* < 5 × 10^−8^ not previously detected by the Global Lipids Genetics Consortium [40] based on <1 mb and r^2^ < 0.001; P, *P*-value; SE, standard error of regression coefficient; SNP, single nucleotide polymorphism.
(XLSX)

S7 Table Correlations between effect estimates of genetic instruments used in multivariable MR analyses. HDL, high-density lipoprotein; LDL, low-density lipoprotein; MR, Mendelian randomisation.
(XLSX)

S8 Table Genetically determined effect estimates from univariable and multivariable MR analyses adjusted for fasting time (LDL, TG, and apolipoprotein B). –Beta, effect estimate; LDL, low-density lipoprotein; MR, Mendelian randomisation; nSNP, number of single nucleotide polymorphisms; P, corresponding *P*-value; SE, standard error of the effect estimate; TG, triglycerides.
(XLSX)

S9 Table Genetically determined effect estimates from univariable and multivariable MR analyses adjusted for fasting time (HDL, apolipoprotein A-I, and apolipoprotein B). Beta, effect estimate; HDL, high-density lipoprotein; MR, Mendelian randomisation; nSNPs, number of single nucleotide polymorphisms; P, corresponding *P*-value; SE, standard error of the effect estimate.
(XLSX)

S10 Table Genetically determined effect estimates from the multivariable MR analysis using the MR-Egger method for LDL, TG, and Apo B. Beta, effect estimate; LDL, low-density lipoprotein; MR, Mendelian randomisation; nSNPs, number of single nucleotide polymorphisms; P, corresponding *P*-value; SE, standard error of the effect estimate; TG, triglycerides.
(XLSX)

S11 Table Genetically determined effect estimates from the multivariable MR analysis using the MR-Egger method for HDL, apolipoprotein A-I, and apolipoprotein B. Beta, effect estimate; HDL, high-density lipoprotein; MR, Mendelian randomisation; nSNPs, number of single nucleotide polymorphisms; P, corresponding *P*-value; SE, standard error of the effect estimate.
(XLSX)

S12 Table Genetically determined effect estimates from univariable and multivariable MR analyses (LDL, HDL, and apolipoprotein A-I). Beta, effect estimate; HDL, high-density lipoprotein; LDL, low-density lipoprotein; MR, Mendelian randomisation; nSNPs, number of single nucleotide polymorphisms; P, corresponding *P*-value; SE, standard error of the effect estimate.
(XLSX)

S13 Table Genetically determined effect estimates from univariable and multivariable MR analyses (TG, HDL, and apolipoprotein A-I). Beta, effect estimate; HDL, high-density lipoprotein; MR, Mendelian randomisation; nSNPs, number of single nucleotide polymorphisms; P, corresponding *P*-value, SE, standard error of the effect estimate; TG, triglycerides.
(XLSX)

S14 Table Effect estimates of 13 loci associated with apolipoprotein B but not the other 4 traits based on *P* < 5 × 10^−8^. apoB, apolipoprotein B; Beta, beta coefficient; BP, base position; CHR, chromosome; eaf, effect allele frequency; p, *P*-value; SE, standard error.
(XLSX)

S1 Text Equation for F-statistic. (DOCX)

## Abbreviations

ACC: American College of Cardiology
AHA: American Heart Association
ANGPLT3/4: angiopoietin-like proteins 3 and 4
CHD: coronary heart disease
CVD: cardiovascular disease
EAS: European Atherosclerosis Society
ESC: European Society of Cardiology
GWAS: genome-wide association study
HDL: high-density lipoprotein
LMM: linear mixed model
MR: Mendelian randomisation
OR: odds ratio
SNP: single nucleotide polymorphism
UKBB: UK Biobank
VLDL: very low-density lipoprotein
