# Supplementary material for: Evaluating the relationship between circulating lipoprotein lipids and apolipoproteins with risk of coronary heart disease: A multivariable Mendelian randomisation analysis
Source: PLoS Med. 2020 Mar 23;17(3):e1003062. doi: 10.1371/journal.pmed.1003062 (PMC7089422; doi:10.1371/journal.pmed.1003062)
Supplement: S1 Fig — Multivariable MR: (A) conventional MR, (B) conventional MR confounded by pleiotropy, (C) multivariable MR. (A) In conventional MR, in the absence of confounding of the genetic instrument and with exclusion restriction, a suitably powered analysis can lead to causal deductions. (B) In this example, SNPs used in the LDL cholesterol instrument also associate with apolipoprotein B (apoB), leading to potential confounding of the estimate of LDL cholesterol and risk of CHD. (C) In multivariable MR, genetic instruments for each of the traits under investigation are used together with the corresponding associations of SNPs with each trait in the analysis. The analysis yields a causal estimate for each trait taking into account the relationship of that trait and the genetic variants with the other traits in the analysis. These figures are schematic representations and should not be interpreted as formal directed acyclic graphs. apoB, apolipoprotein B; CHD, coronary heart disease; CI, confidence interval; HDL, high-density lipoprotein; LDL, low-density lipoprotein; MR, Mendelian randomisation; SNP, single nucleotide polymorphism. (PDF) [file pmed.1003062.s002.pdf]

S1 Fig: Multivariable Mendelian randomization

(A) Conventional Mendelian randomization

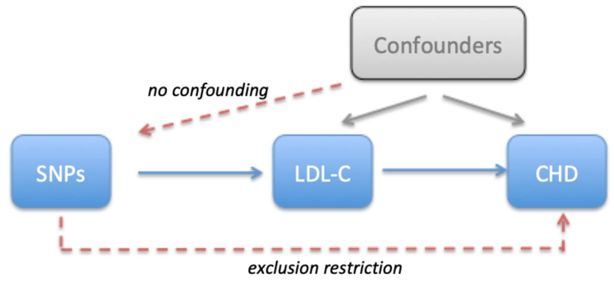

In conventional Mendelian randomization, in the absence of confounding of the genetic instrument and with exclusion restriction, a suitably powered analysis can lead to causal deductions.

(B) Conventional Mendelian randomization confounded by pleiotropy

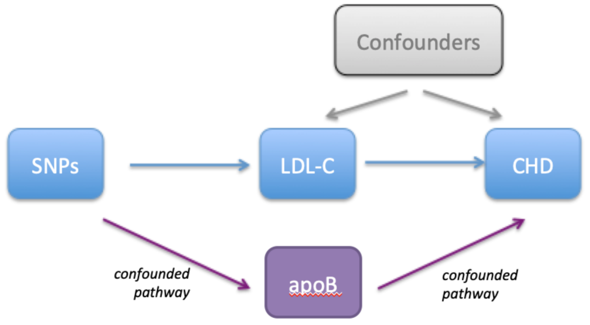

In this example, SNPs used in the LDL cholesterol instrument also associate with apolipoprotein B (apoB), leading to potential confounding of the estimate of LDL cholesterol and risk of CHD.

(C) Multivariable Mendelian randomization

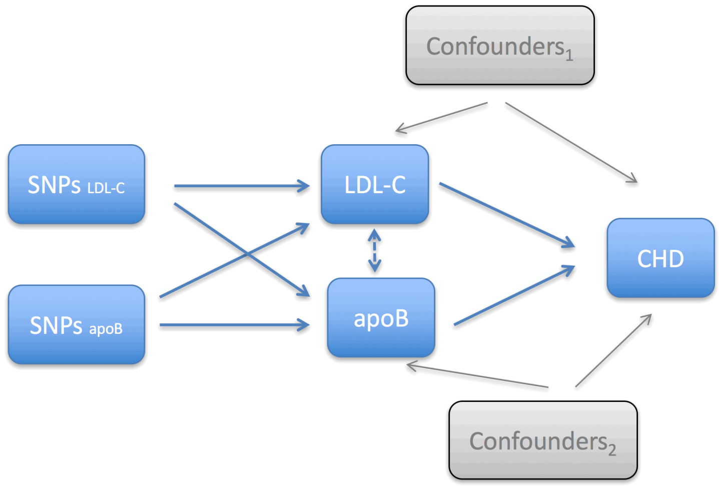

In multivariable MR, genetic instruments for each of the traits under investigation are used together with the corresponding associations of SNPs with each trait in the analysis. The analysis yields a causal estimate for each trait taking into account the relationship of that trait and the genetic variants with the other traits in the analysis.

These figures are schematic representations and should not be interpreted as formal directed acyclic graphs. apoB = apolipoprotein B, CHD = coronary heart disease, CI = confidence interval, HDL = high-density lipoprotein, LDL = low-density lipoprotein, SNP: single nucleotide polymorphism.
