# Supplementary material for: Evaluating the relationship between circulating lipoprotein lipids and apolipoproteins with risk of coronary heart disease: A multivariable Mendelian randomisation analysis
Source: PLoS Med. 2020 Mar 23;17(3):e1003062. doi: 10.1371/journal.pmed.1003062 (PMC7089422; doi:10.1371/journal.pmed.1003062)
Supplement: S2 Fig — Effect estimates are ORs of CHD per 1-standard-deviation–higher genetically instrumented trait, using a range of univariable MR approaches (see Methods for further details). Plot generated using Stata SE 13.1 (StataCorp). CHD, coronary heart disease; CI, confidence interval; HDL, high-density lipoprotein; LDL, low-density lipoprotein; MR, Mendelian randomisation; nSNPs, number of single nucleotide polymorphisms; OR, odds ratio. (PDF) [file pmed.1003062.s003.pdf]

S2 Fig: Univariable Mendelian randomization estimates for individual lipid and apolipoprotein traits

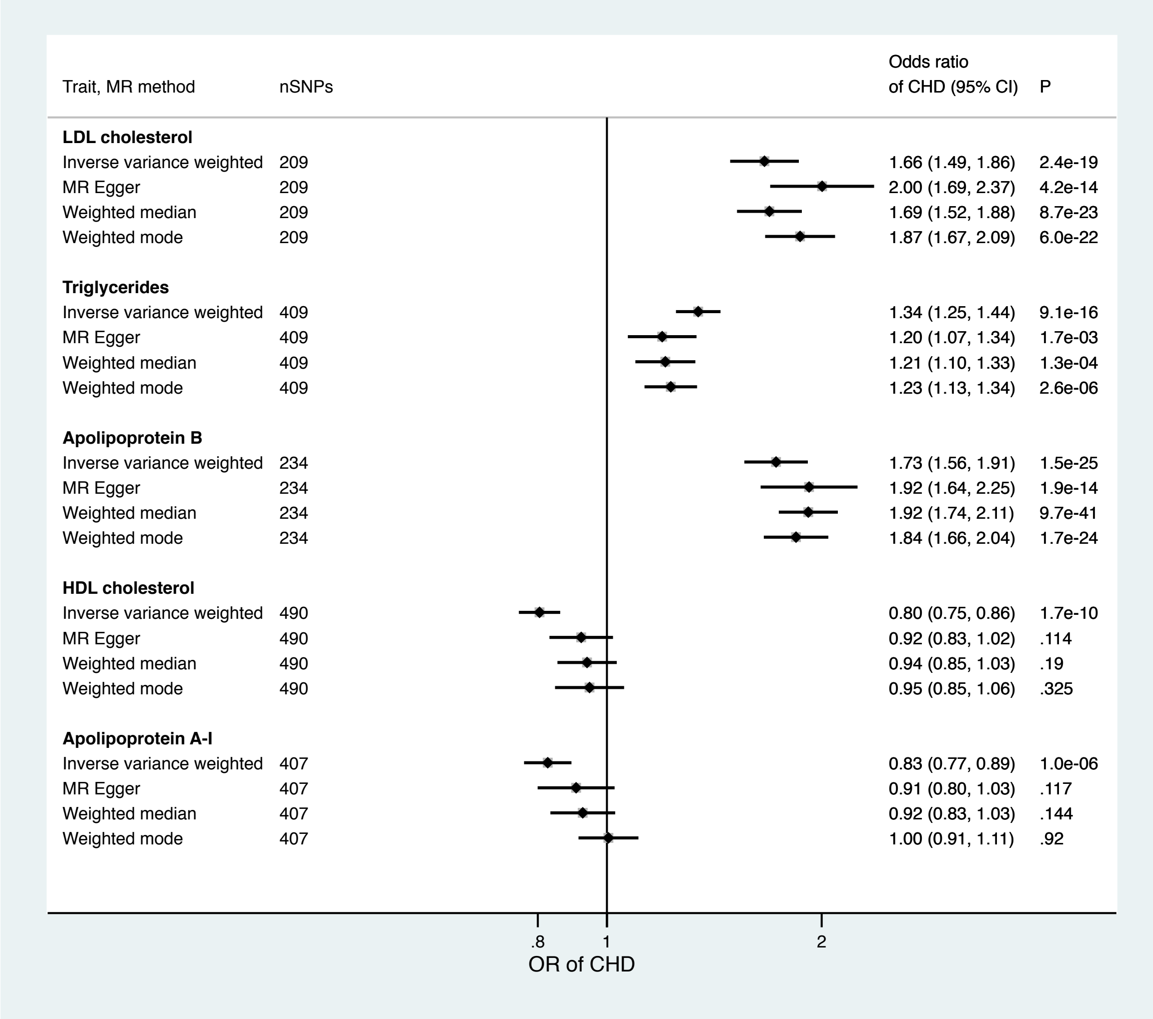

Legend: Effect estimates are odds ratios of coronary heart disease (CHD) per 1 standard deviation higher genetically instrumented trait, using a range of univariable Mendelian randomization approaches (see Methods for further details). Plot generated using Stata SE 13.1 (StataCorp). CI = confidence interval, HDL = high-density lipoprotein, LDL = low-density lipoprotein, MR = Mendelian randomization, OR = odds ratio, nSNPs = number of single nucleotide polymorphisms.
