# Supplementary material for: Evaluating the relationship between circulating lipoprotein lipids and apolipoproteins with risk of coronary heart disease: A multivariable Mendelian randomisation analysis
Source: PLoS Med. 2020 Mar 23;17(3):e1003062. doi: 10.1371/journal.pmed.1003062 (PMC7089422; doi:10.1371/journal.pmed.1003062)
Supplement: S1 Text — (DOCX) [file pmed.1003062.s018.docx]

**S1 Text: Equation for F-statistic**

We characterized instrument strengths in the univariable MR setting by generating the mean F-statistic, using the approximation described by Bowden et al[1]:

$$F_{j}=\frac{\gamma_{j}^{2}}{\sigma_{Xj}^{2}}$$

where γ_j_ is the SNP-exposure association and σ_Xj_ is the standard deviation for the SNP-exposure association for variant j.

For the multivariable estimate, we generated the conditional F-statistic [2] as described in the article by Sanderson and colleagues. [3]

References to S1 Text:

1. Bowden J, Del Greco MF, Minelli C, Davey Smith G, Sheehan NA, Thompson JR. Assessing the suitability of summary data for two-sample Mendelian randomization analyses using MR-Egger regression: the role of the I2 statistic. Int J Epidemiol. 2016. doi: 10.1093/ije/dyw220. PubMed PMID: 27616674.

2. Sanderson E, Spiller W, Bowden J. Testing and Correcting for Weak Instruments in Two-sample Summary Data Multivariable Mendelian Randomisation. In preparation. 2019.

3. Sanderson E, Davey Smith G, Windmeijer F, Bowden J. An examination of multivariable Mendelian randomization in the single sample and two-sample summary data settings. Int J Epidemiol. 2019;48(3).
